# Cases of Oral Surgery

**Published:** 1908-01-15

**Authors:** Charles H. Oakman

**Affiliations:** Detroit


### CASES OF ORAL SURGERY.

BY CHARLES H. OAKMAN, D.D.S., M.D., DETROIT.

Read before the Southwestern Michigan Dental Society, at Battle Creek, April 10, 1907.

The removal of impacted lower third molars, of the second or third degree, require careful consideration. If the tooth be invisible, a skiagraph of the parts is a good thing to secure in most cases. I prefer the use of local anesthetics to be injected slowly into the parts. If the tooth is entirely covered by gum tissue, an incision should be made longitudinally in the gum, exposing the tooth as much as possible, the bone drilled on buccal surface until roots of tooth are well exposed. Drilling should never be done lingually, as paralysis may result if the inferior dental nerve be injured, which I did once in a very severe case, and it caused me much anxiety. The same may be caused if the bone be splintered and a piece forced down next to the nerve. Forceps should never be used in these cases until the tooth be loosened and can readily be raised from its bed without any great effort. Murderous attempts have been made to remove these molars with forceps, causing even fracture of the jaw in many cases, and other equally injurious results.

A thin strong blade of an elevator should be passed down between the second and third molars, using the second molar as a fulcrum. Care should be taken not to exert too much force and displace second molar. The distal portion of crown of second molar may be cut, in some cases; I have never found it necessary. In cases of the second and third degrees never but one tooth should be removed at a sitting. The wound should be packed with gauze, and treated antiseptically for several days. Patient supplied with medicine to control after pains. Application of hot water bag is of great service. Many so-called impacted molars are removed with ease, and can properly be called "pseudo impactions." Where the bone is massive and of a very inorganic nature considerable skill is sometimes required in handling these cases. Many times nothing short of profound anesthesia will suffice. The patient should be brought well under the influence, as there is less damage from shock.

History of a case: Mrs. W., aged 30 years. Had two impacted lower third molars, one of the second and the other of the third degree. She was anxious for the removal of both. I advised but one at a time; she insisted, and against my better judgment I removed both teeth. Chloroform in this case was used, and both teeth removed. For about two weeks the patient suffered from a severe trismus, scarcely the handle of a teaspoon could be forced between teeth, and rectal feeding had to be resorted to. The corner of the mouth was depressed and there was a partial facial paralysis. Drouling and many other disagreeable features were present. Patient regained her normal condition after several weeks, as her general health improved.

### DISEASES OF ANTRUM.

*Tumors.*—Malignant tumors often begin in the antrum, such as sarcoma, and a very rapidly growing form of epithelioma.

A sarcoma of upper jaw may at first cause symptoms resembling toothache or neuralgia. Epithelioma may also simulate caries or necrosis; these symptoms should always place the surgeon on his guard. My personal observation regarding the prognosis of cases of malignant growths has been that they terminated by death, after running a period of seven or eight months, unless an *early radical* operation be performed.

In a case of sarcoma, the prognosis largely depends on the type of the histologic structure of the tumor. A giant celled sarcoma, if completely removed, seldom recurs. Metastatic growths seldom appear after operation, whereas in round and spindle celled sarcoma, *although* the original growth be apparently removed, secondary growths nearly always reappear. A careful microscopical examination of the tumor should always be made.

History of a case—No. 1: Mr. M., 45 years of age, laboring man, sent to me for examination regarding condition of antrum. Patient complained of pain in the region of antrum, malaise, anarexia, and discharge from nose, etc. Patient showed a marked cachexia. Examination of the external region of antrum showed bulging. The circulation of that region was impaired, and it was evident an operation was necessary. As the teeth were loose, the bicuspids and first molar were extracted and a portion of the floor of the antrum removed. It was found to be filled with a new growth, a portion of which was removed and immediately examined. The report stated it to be an epithelioma, the removal of which would necessitate the resection of upper maxilla. This the patient refused to consent to. Found it necessary to remove the cauliflower growth, which grew so rapidly that in ten days to two weeks the orifice of cavity would be occluded. Patient absolutely refusing radical operation, and X-Ray treatment proving futile. On account of the progress of the growth affecting his personal appearance the patient would not venture to appear on the street, and the case terminated in an agonizing death, four months after his first appearance at office.

Mr. A., merchant, 50 years of age. Complained of a pain to right of median line, just at vestibule of the nose. Two physicians examined him and agreed that there must be some exciting cause at point mentioned. I met the medical men in consultation, but took issue with them on their diagnosis. They agreed to take the brunt of criticism, should I operate, and operation prove a failure. Reluctantly, I operated; kept parts packed with iodoform gauze for a week, daily examinations and a few probings. The result was negative, hence there was no relief to patient. A couple of months later he returned and asked me to do for him what I might in order to give him relief. I was firmly of the belief that the antrum was involved. Made an exploratory opening, pus flowed freely. At the hospital next day a radical operation was done. Outer plate of superior maxillary was distended, and on deep palpation the parts crackled like parchment. The case was edentulous. Removed the floor of the antrum with little effort, as the parts were necrotic, the mucous tissue was curetted, and antrum packed with iodoform gauze. In ten days fitted a plate with a protuberance just the size of the orifice of wound. Patient is wearing it with comfort, and has no difficulty in masticating. This case presented a well defined septum within the antrum. Patient's health was greatly impaired on account of septic absorption. This case was undoubtedly of several years' standing. He is now enjoying good health. ht is eighteen months since his operation, and his general health is quite good, although he was never robust. The distention of the outer plate of both maxillary bones was very marked, so much so that it was a question whether or not it was a bilateral case. It proved, however, to be unilateral.

Mr. R., aged 53 years, occupation capitalist. Called on me for examination of his case. He had spent the year abroad with his wife and family, in the hope of regaining his nervous equilibrium. On his return journey, while in London, he was taken ill with pain in the frontal and maxillary sinuses, on right side, which caused him great suffering. He had been examined by a physician and dentist, who failed to diagnose his case. He lay in a comatose state for several days; in the course of a week he was able to be up again, when he took passage on a steamer for home. The second day out he lapsed into a comatose state. Later the ship's surgeon quieted his pain by the use of opiates. After reaching New York he had a tooth extracted; this gave but slight relief. The following day I saw him. I found a copious discharge of pus from the nose. The odor was plainly discernible at speaking distance. His handkerchiefs were saturated with odorous pus.

Using a local anesthetic, I drilled into the tooth socket and secured a liberal flow of pus, found the floor of the antrum necrotic. A few days after a radical operation followed at hospital.

Removed all the teeth, posterior to the cuspid necrotic bone and mucous tissue of the antrum. In ten days' time fitted an appliance attached to a rubber plate. Two teeth were absent on the opposite side, which made the plate more secure.

In two weeks' time he became thoroughly accustomed to his appliance, and he was able to go South for the remainder of the winter. Saw the patient last week on his return home. He is in excellent health and spirits, his recovery being complete.

Mr. R. H., 40 years, a printer by occupation. Was sent by his physician to me for treatment of an aggravated case of pyorrhea; practically all of his teeth were affected, especially the molars and bicuspids on the upper right side. After two treatments for the removal of calcareous deposits from the roots, I explored the buccal sockets of the first molar and found I could easily insert a probe into the antrum. A slight amount of odoriferous pus percolated into the mouth. The next day I concluded to operate on antrum and made a liberal incision over the roots of bicuspids and first molar, the tissue was raised up with periosteotome, and bone drilled the length of the incision, securing an opening big enough to feel with finger. I then curetted the mucous tissue of antrum, irrigated and packed with iodoform gauze, dilating the orifice of wound as much as possible with the gauze, in order to facilitate the insertion of a small electric light and secure a view of the parts at some future time. After a week's irrigation and tamponing, I fitted a vulcanized rubber plug large enough to keep wound open. After a month of daily irrigation of boric acid and sodium bicarbonate solution, I concluded to allow wound to heal, which it did rapidly after scarifying the edges.

First. The *subjective* symptoms were general malaise.

Second. On arising in the morning a bad taste was noticeable.

Third. It was generally noon before the patient would begin to take interest in things without great effort.

Fourth. Experienced no pus coming from the nose.

Fifth. Had occipital and frontal pains. At no time were they severe.

Sixth. Constipation.

Seventh. Loss of the sense of taste.

Eighth. Despondent at times.

*Treatment* (*Systemic*).—Calomel in fractional doses, A. S. & B. pills, cascara, saline cathartics, castor oil and tonics.

The *interesting* part of this case is the quick return of the sense of taste. Two days after operation he informed me that he could taste his food, stated there was a great change taking place somehow. Coffee no longer seemed the same as water to him. His taste seemed to improve immediately.

Patient became enthusiastic from the fact that his taste, which had been lost for a few years, had been restored to him. The malaise ceased immediately, and his general condition improved in every way. Now when he arises in the morning there is a great difference; he is active, bright and cheerful, and expresses himself that he has taken a new lease of life. I know of no case in the literature where the taste returned as quickly as in this.

*Antral Case.*—Mr. W., aged 29 years. Occupation, cigar-maker. I was called in to operate on the upper left jaw. Diagnosis, empyema of antrum, with necrosis; carious third molar previously extracted on account of pain, allowing pus to come through the tooth sockets.

Bicuspids and first molar were loose and a necrotic condition plainly discernible; loose teeth were extracted, diseased bone and mucous tissue of antrum were curetted, the wound was packed tightly with iodoform gauze to arrest a profuse hemorrhage. After eight days patient returned to his home temporarily; he did well for five days, when he contracted a septic condition of the upper part of left lung, no doubt induced by some of the septic material being inspired into the lung. For a month patient was in a precarious condition; pus was present in the antrum and continued to menace the patient's health. The antrum was irrigated and packed several times a day. Temperature ran up to 104½, pulse 120-140. He had become emaciated. The cavitv in lung had become larger, and it seemed for a time that he might not be able to withstand the ordeal.

Echafolta was administered with good results, it regulated the pulse and reduced his temperature. I feared an anesthetic of ether in this case, thinking it would cause a case of pneumonia, so waited for the lung condition to clear up. As it did not and his temperature did not at this time exceed 100 degrees, I had him brought to Detroit for a second operation. On entering hospital temperature was 101, pulse 130. Ten days lapsed and no change had taken place, except a slight drop in temperature from the first day he entered hospital, as the effort of a 100-mile travel and excitement incident to the trip sent it up slightly. His chest was examined several times by a specialist; the pus could not be aspirated on account of the position, scapula and clavicle being in the way. Several examinations were made of the sputum; but no T. B.'s found, concluded to again operate, as it appeared that the septic antrum was still the exciting cause.

Chloroform was administered and operation done as quickly as possible. Second molar, the only tooth present, was extracted, and bone cut out from cuspid to distal end of antrum, and bone removed as far as molar process. Portion of palatal bone also removed and antrum was tightly packed with iodoform gauze. Day after operation, temperature 97, pulse 88, a drop in temperature of 4 degrees, pulse 32.

His appetite was good most of the time and feeding was carried on to the limit. Bowels were carefully watched and tonics administered. The patient has left the hospital, but will need careful attention for some time.

*Tubercular Case.*—M. M., child 9 years of age, was sent by her dentist to me for treatment of a swelling in the region of the first lower right molar, which had been extracted, but the pain and swelling continued, pus had nearly burrowed to the outer surface of neck, so far, indeed, that it was necessary to make an incision in the skin about an inch from the free border of the jaw; a second incision was made on the inside of the cheek, and a curved dressing forceps passed through, connecting both wounds. Some bone was drilled out and parts curetted and wound packed tightly with iodoform gauze . The pus discharging from this wound had a peculiar caseous appearance, and on examination by the bacteriologist it was found to contain tubercle bacillus.

Drainage was kept up for several weeks, when the parts healed. The child's temperature was from 99.2 to 101 and continued so for some weeks; after the wound healed, temperature was normal. It is about two and one-half years, with no recurrence. I thought it would be necessary to remove the cervical glands, but see no occasion for it now.

*Case of Hemorrhage.*—Mr. J. G., aged 40 years, occupation, saloonkeeper. Had been affected with frequent hemorrhages from the gums, which lasted several months. Was confined to his bed about half of the time. He was always a hard drinker, and apparently could not refrain from it. His physician referred the case to me. The hemorrhage was not from the gums alone, but apparently from most of the mucous tissue of the body; bloody stools were frequent. He was of a very plethoric temperament, and had endocarditis, nephritis and luetic taint. Had him come to the office and removed all calculi and other irritants from the teeth prescribed astringent mouth-washes, chlorid of zinc, tannic acid, alum and other astringents. Nightly, for several weeks, he would lose from one to six ounces of blood, and eventually became very weak. I applied *Suprarenal* gland extract in powder form and secured a good result, which lasted 24 hours. Continued it the next day with fair success as far as hemorrhages was concerned. He had a bad reaction, due to the high blood pressure, induced by the absorption of the Suprarenal gland extract. Reduced the amount of powder, but for steady use I had to seek something else. Came across an article written by one of New York's gynecologists, citing an instance where, in a case of endometritis, he had used, with success, Thyroid gland extract to control the hemorrhage. This prompted me to try the Thyroid extract in this case. Started with three tablets a day for three days, and then four a day for four days, then reduced to three daily, occasionally two a day. Ten days after taking these tablets there was no sign of hemorrhage, patient continued to improve slowly; ebout eight days from arrest of hemorrhage he was able to go downtown although very weak.

After his second visit downtown, he was induced to take a few drinks of whisky, and a few more; when he had to be brought home suffering from the effects of this indulgence. I called next day, found him in bed with renewed hemorrhage. Could not account for the change, when he had been doing so well. His wife quietly told me the circumstances. In a few days hemorrhage again ceased to some extent. In the course of a few days he was able to be out again, and made a few trips to business. He again fell by the wayside, which brought on hemorrhage. After a strong admonition not to use alcohol except it be prescribed, which I knew would not be likely, I concluded to stay by the case a little longer, although greatly annoyed by his actions. Several other escapades, and I refused to treat him further, as I learned that he was taking alcohol without my knowledge. It was a question whether hemorrhage, nephritis with its attendant sclerosis, paresis, or endocarditis would be the most important factor in ending his sufferings. As I did not attend him toward the last, I am not familiar with the latter condition. Death occurred four months later from a complication of the above diseases.

*Excision of Lower Jaw.*—Mrs. W., aged 34, years, had necrosis of lower jaw; the etiology of which was probably strepto coccus infection; the gums had been lanced repeatedly but of no avail. Several external openings were made under chin, but failed to give any permanent relief. When the case came into my hands patient had been sick in bed about six weeks, temperature 101 to 104, and in a very weakened condition; took but a glance to see that the jaw was severely necrotic. Patient's face was greatly distorted. Operated the next day after her arrival at hospital. United the small incisions under chin into one large one, inserted probe along lower border of jaw on either side as far as the ramus. Pus and debris were curetted out in large quantities, the parts irrigated and wound packed with iodoform gauze from one ramus to the other. Post-operative conditions caused considerable anxiety. Next morning patient's eyes were closed, and face was a sight to behold, temperature 103, pulse 160. For three days the patient was in a very serious condition. Three weeks later swelling was reduced when a second operation was done, and the lower left jaw from ramus to median line was removed. As it was completely necrotic, the bone was removed from within the mouth and from the wound under the chin, exfoliation was taking place of a large piece of bone on the lingual surface near ramus and forward as far as the bicuspids. On the opposite side the wound inside of mouth was the full length of jaw; it had been kept open for five weeks, trusting that in time there might be an exfoliation. When the wound closed up a large swelling occurred, which involved the neck as far as the clavicle, also the mastoid portion as far back as the middle of the neck. The pus was on the verge of burrowing through the outer surface of the skin. Tried not to open on the surface, but found nothing else would do, so an incision of five inches was made, extending past the lobe of the ear. About two inches of bone removed intact and parts curetted. Nothing left but thin ridge of bone and a few teeth, which may be removed easily. The wound is almost healed.

*Sarcoma.*—Miss B. S., aged 14 years, was sent by her dentist to me for examination and treatment. He had extracted a carious lower right bicuspid, which failed to yield to treatment. There was a considerable swelling, the tumor extending from median line to distal of first molar, about size of small hen's egg. On palpation the tumor was soft and had the appearance of a cyst. Local anesthetic was injected into the mucous tissue, and incision made the length of tumor. Found it very vascular, and the whole thickness of bone was affected until the floor of the mouth was reached. The bone was drilled out and parts curetted. By this time I was satisfied that I had on hand something of a serious nature. Sent tissue to pathologist who pronounced it a spindle cell sarcoma of a very malignant type. After this report, I concluded to do a more radical operation and remove the jaw as far as ramus and to the left of median line.

Her parents became alarmed on hearing it was a cancer, and sought advice from many sources. The concensus of opinion was to operate. The thought of disfigurement was naturally trying to her family, as they had a cousin who had a cancer of the hip joint, which was inoperable, and he was sent to Dr. Cooley of New York, for the streptococcus and prodigiosus toxin treatment. His parents were informed he probably would not live six months when he left Detroit. The treatment was given him in New York the greater part of eleven months. He has gained ten pounds and was able to walk without crutches.

My little patient's family knowing this, thought the same treatment would prove beneficial to her, so she spent several weeks under Dr. Cooley's care. The treatments were at first very severe. She is still taking them, but the time is too short to give any positive results. Dr. Cooley, when he first saw the case, rather favored operation, as Dr. Cooley prefers to employ the toxin treatment in inoperable cases. I am watching the case with deep interest.

